# Interleukin-1β Modulates Synaptic Transmission and Synaptic Plasticity During the Acute Phase of Sepsis in the Senescence-Accelerated Mouse Hippocampus

**DOI:** 10.3389/fnagi.2021.637703

**Published:** 2021-02-10

**Authors:** Koji Hoshino, Yuka Uchinami, Yosuke Uchida, Hitoshi Saito, Yuji Morimoto

**Affiliations:** Department of Anesthesiology, Hokkaido University Hospital, Sapporo, Japan

**Keywords:** sepsis-associated encephalopathy, synaptic plasticity, microglia, interleukin-1β, neuroinflammation

## Abstract

**Background:**

Aging and pre-existing cognitive impairment are considered to be independent risk factors for sepsis-associated encephalopathy. This study aimed to investigate the manner in which aging and pre-existing cognitive dysfunction modified neuroinflammation, synaptic plasticity, and basal synaptic transmission during the acute phase of sepsis using Senescence-Accelerated Mice Prone 8 (SAMP8) and Senescence-Accelerated Resistant Mice 1 (SAMR1).

**Methods:**

We used 6-month-old SAMP8 and SAMR1. Sepsis was induced using cecal ligation and puncture (CLP). The animal’s hippocampi and blood were collected for subsequent investigations 24 h after surgery.

**Results:**

Long-term potentiation (LTP) was impaired in the Shaffer-collateral (SC)-CA1 pathway of the hippocampus in SAMP8 without surgery compared to the age-matched SAMR1, which was reflective of cognitive dysfunction in SAMP8. CLP impaired the SC-CA1 LTP in SAMR1 compared to the sham-operated controls, but not in SAMP8. Moreover, CLP decreased the input-output curve and increased the paired-pulse ratio in SAMP8, suggesting the reduced probability of basal synaptic transmission due to sepsis. Immunohistochemical analysis revealed that CLP elevated IL-1β levels, especially in the hippocampi of SAMP8 with microglial activation. *In vivo* peripheral IL-1 receptor antagonist (IL-1ra) administration in the septic SAMP8 revealed that the neuroinflammation was not correlated with the peripheral elevation of IL-1β. *Ex vivo* IL-1ra administration to the hippocampus ameliorated LTP impairment in SAMR1 and the reduction in basal transmission in SAMP8 after sepsis.

**Conclusions:**

The mechanism of the modulation of synaptic transmission and synaptic plasticity by the acute stage of sepsis differed between SAMR1 and SAMP8. These changes were related to centrally derived IL-1 receptor-mediated signaling and were accompanied by microglial activation, especially in SAMP8.

## Introduction

Sepsis causes multiple-organ dysfunction via dysregulation of the host response to infection, and the brain is often one of the first organs affected by sepsis (Pytel and Alexander, 2009). Sepsis-associated encephalopathy (SAE) is defined as diffuse brain dysfunction accompanied by sepsis, in the absence of direct central nervous system infection, which manifests as cognitive dysfunction, especially learning and memory disabilities (Gofton and Young, 2012; Widmann and Heneka, 2014). SAE can be an independent risk factor for mortality (Sprung et al., 1990). Although the pathophysiological mechanism of SAE is thought to be multi-factorial, neuroinflammation-induced functional neuronal changes involving microglial activation in different regions of the brain, including the hippocampus, is the predominant process (Robba et al., 2018; Barichello et al., 2019). In fact, we previously reported that long-term potentiation (LTP) in the mouse hippocampus, which could be regarded as the cellular basis of learning and memory (Bliss and Collingridge, 1993), was impaired via mechanisms associated with microglial activation and interleukin (IL)-1β activity during the acute phase of sepsis (Hoshino et al., 2017b).

Sonneville et al. (2017) recently reported that aging and pre-existing cognitive impairment were independent risk factors for SAE (Sonneville et al., 2017). Although several studies have demonstrated the relationship between neuroinflammation and cognitive dysfunction caused by sepsis using healthy aging animals (Barrientos et al., 2015), to the best of our knowledge, no study has explored the influence of sepsis on synaptic function using an animal model of aging combined with cognitive dysfunction.

The Senescence-Accelerated Mouse (SAM) strains have been successfully developed by selective inbreeding of the AKR/J strain of mice, and numerous studies using SAM models have been carried out in various fields of aging science since 1981 (Takeda et al., 1981). SAM Prone 8 (SAMP8) is an experimental animal model that exhibits rapid senescence and age-related cognitive dysfunction (Takeda, 2009), while the Senescence-Accelerated Resistant Mouse 1 (SAMR1) has normal aging characteristics and is used as the control. The characteristics of the SAMP8 are similar, but not entirely identical, to those of the Alzheimer’s disease brain in humans, e.g., the deposition of β-amyloid protein in the different regions of the brain, including the hippocampus (Takeda, 2009). It is a well-known fact that systemic immune challenges exacerbate cognitive decline in patients who are already affected by chronic neurodegenerative diseases such as Alzheimer’s disease (Perry et al., 2007). Therefore, experiments with SAMP8 are thought to be useful for gaining insight into the pathophysiology of SAE concomitant with cognitive dysfunction among the elderly.

The aim of our study was to investigate how aging and pre-existing cognitive dysfunction affect neuroinflammation, synaptic plasticity, and basal synaptic transmission during the acute phase of sepsis using SAMP8 and SAMR1 animal models.

## Materials and Methods

### Animals and Sepsis Model

Male SAMP8/Ta Slc and SAMR1/Ta Slc (6 months old) were purchased from SLC (SLC Japan Inc., Shizuoka, Japan). The mice were housed at a controlled temperature with a 12-h:12-h light-dark cycle and were allowed access to food and water *ad libitum*. All experiments were performed according to the guidelines for the care and use of laboratory animals of Hokkaido University, and were approved by the Animal Care and Use Committee of Hokkaido University (No. 17-0053).

The cecal ligation and puncture (CLP) procedure was first developed by Chaudry et al. (1979), Hubbard et al. (2005) and is considered to be the gold standard for sepsis research, owing to the reproducibility of its hemodynamic and metabolic changes. We have described modification to this method in a previous study (Hoshino et al., 2017b). A 1-cm long midline abdominal incision was performed after the animals were administered deep anesthesia with pentobarbital (50 mg/kg, intraperitoneally) and butorphanol (5 mg/kg, intraperitoneally). The cecum was carefully mobilized and subsequently ligated with 3-0 silk sutures, distal to the ileocecal valve. The cecum was perforated once with a 21-G needle and squeezed to extrude some feces into the peritoneal cavity. The abdomen was closed in two layers and the mouse was resuscitated postoperatively with a subcutaneous injection of Ringer’s lactate solution (30 mL/kg). The entire procedure was completed within 10 min. Sham-operated mice underwent the same surgical procedures without CLP.

### Behavioral Measurement (Trace-Fear Conditioning Test)

We conducted the trace-fear conditioning test, which reflects hippocampal-dependent memory, without surgery (Phillips and Ledoux, 1992), to confirm the cognitive dysfunction in 6-month-old SAMP8 in comparison with SAMR1. Each mouse was placed in a commercially available conditioning chamber (Panlab Inc., Barcelona, Spain), and underwent a training session consisting of eight cycles with a 0.8-mA foot shock interspersed by a randomly selected 1–4 min interval. The mice were returned to the same chamber 3 days after training and underwent the 4-min memory retention test. Subsequently, the behavior of mice was analyzed by an investigator who was blinded to the mouse species, and the duration of the freezing behavior was measured. Freezing was defined as the lack of movement, except for breathing. The freezing behavior was expressed as a percentage of the 4-min retention period.

### Endotoxin Measurement

We measured the endotoxin concentration in blood 24 h after surgery to confirm the validity of the sepsis model, irrespective of the mouse species. Approximately 500 μL of blood was collected from the inferior vena cava of the mice after the administration of deep anesthesia (with pentobarbital and butorphanol, as described above). Endotoxin concentration was measured with a toxinometer (MT-6500; Wako, Osaka, Japan).

### Pro-inflammatory Cytokine Measurement in the Hippocampus and Plasma

Blood samples were collected as described above, 24 h after surgery, and each sample was centrifuged at 2,000 rpm for 20 min. The hippocampus was harvested and weighed, quickly followed by homogenization in tissue lysis buffer and centrifugation at 3,000 rpm for 5 min. The plasma samples and homogenized hippocampal supernate were stored at −80°C for further analysis. The levels of IL-1β, tumor necrosis factor (TNF)-α, and IL-6 were measured using commercially available enzyme-linked immunosorbent assay kits (R&D Systems, Minneapolis, MI, United States) according to the manufacturer’s instructions.

### Immunohistochemistry

Tissue preparation was based on our previously reported method (Saito et al., 2018). The mice underwent transcardial perfusion of 0.1 M phosphate-buffered saline, followed by cold 4% paraformaldehyde in 0.1 M phosphate buffer 24 h after surgery. The brains were harvested and immersed overnight, first in the same fixative, second, in 20% sucrose for 24 h, and finally, in 30% sucrose for another 24 h. Subsequently, the brains were freeze-embedded in optimal cutting temperature compound (Tissue-Tek O.C.T.; Sakura Finetek, Japan), and cut sequentially into 30 μm-thick coronal sections with a cryostat (CM1850; Leica Biosystems, Wetzkar, Germany) and mounted on a glass slide. The microglia were labeled using rabbit polyclonal antibody, anti-Iba-1, at a concentration of 1:1000 (Wako, Osaka, Japan). The immunoreactivity of Iba-1 was visualized using a Rabbit specific HRP/DAB (ABC) Detection IHC kit (ab64261; Abcam, Cambridge, United Kingdom) in accordance with the manufacturer’s instruction. Negative controls were created for all mice specimens by not incubating them with the primary antibody. Immunohistochemical photomicrographs were obtained with a microscope (BIOREVO BZ-9000; KEYENCE, Osaka, Japan). Immunohistochemical staining was assessed by an observer who was blinded to the group using two randomly selected slices from the CA1 region of each mouse according to the following four-point categorical scale (Colburn et al., 1997): score 0 = unperturbed microglia extensively ramified, well-spaced, wispy appearance; score 1 = microglia still ramified, less area between individual microglia; score 2 = microglia less ramified, increased density of microglial cells, occasionally overlapping; score 3 = microglia exhibit short bold projections, densely arranged/extensive overlapping.

### Electrophysiology

We have described the method of slice preparation in a previous study (Hoshino et al., 2017a). The mice were decapitated after deep anesthesia with isoflurane and the brain was dissected in an ice-cold sucrose solution composed of the following (in mM): 40 NaCl, 25 NaHCO_3_, 10 glucose, 150 sucrose, 4 KCl, 1.25 NaH_2_PO_4_, 0.5 CaCl_2_, and 7 MgCl_2_. Transverse hippocampal slices were prepared (300-μm thick) with a linear slicer (PRO7; DOSAKA-EM, Kyoto, Japan). Subsequently, the sucrose-containing solution was replaced with artificial cerebrospinal fluid (ACSF) containing the following (in mM): 127 NaCl, 1.5 KCl, 1.2 KH_2_PO_4_, 26 NaHCO_3_, 10 glucose, 2.4 CaCl_2_, and 1.3 MgCl_2_. The slices were incubated for 30 min at 30°C and for another 30 min at 25°C in an interface-type chamber with saturated 95% O_2_ and 5% CO_2_. The slices were transferred to an observation chamber after incubation, and continuously superfused at 2 mL/min with ACSF saturated with 95% O_2_ and 5% CO_2_. A tungsten concentric bipolar stimulating electrode and glass recording electrode with 10-μm diameter tips filled with ACSF were placed in the stratum radiatum of the CA1 region in the hippocampus for the extracellular recordings. Electrical stimuli (duration: 200 ms, intensity <500 μA) were delivered every 10 s and the resultant excitatory postsynaptic potentials (EPSPs) were recorded. The intensity was increased to the threshold of action potential generation to induce maximum LTP. LTP was induced with the high frequency stimulation (HFS) protocol (three-trains of 10-Hz stimulation for 1 s at 20 s intervals) after confirming the stability of the baseline EPSP amplitude for at least 20 min. We compared the EPSP amplitudes expressed 60 min after HFS with the baseline, as described in previous studies (Ferguson et al., 2004; Zivkovic et al., 2015). We generated average input-output curves of the Schaffer collateral (SC)-CA1 pathway for basal transmission analysis. Data were normalized to the maximal EPSP and stimulus intensity range in each experiment to enable inter-slice comparison, as previously reported (Fleck et al., 2000). Moreover, we measured the paired-pulse ratio in each slice, which was considered to be a facet of short-term synaptic plasticity. Paired-pulse stimuli with a 50-ms interval were applied at half the maximal intensity, and EPSP_2_/EPSP_1_ was recorded after the stabilization of the EPSP amplitude. All recordings were made at 25°C using a MEG-5200 amplifier (NIHON KOHDEN, Tokyo, Japan) and LabChart 7 software (AD Instruments, Colorado Springs, CO, United States).

### Drug Administration

IL-1ra (Anakinra; Amgen Inc., Thousand Oaks, CA, United States) was administered subcutaneously at a dose of 100 mg/kg, immediately before surgery in the *in vivo* experiment, according to a previous study (Terrando et al., 2010). The vehicle-treated mice received an injection of an equal volume normal saline. IL-1ra (100 mg/mL) was delivered to the hippocampal slice for at least 20 min for the *ex vivo* experiment, which was enough to block the IL-1 receptor, as per a previous study (Schneider et al., 1998).

### Statistical Analysis

Data were presented as the mean ± standard deviation (SD) and analyzed using Origin Pro 2020b (Origin Lab Co., Northampton, MA, United States). After confirming normality using Shapiro-Wilk test, the two groups were compared using Student’s *t* test. The two-way analysis of variance was used to compare continuous data from multiple groups, followed by Tukey’s *post hoc* test. Categorical data from multiple groups were compared using the Kruskal-Wallis test, followed by Dunn’s *post hoc* test. *P*-values <0.05 were considered statistically significant.

## Results

### Validation of the Mouse Model of Cognitive Dysfunction and Sepsis

**FIGURE 1:**
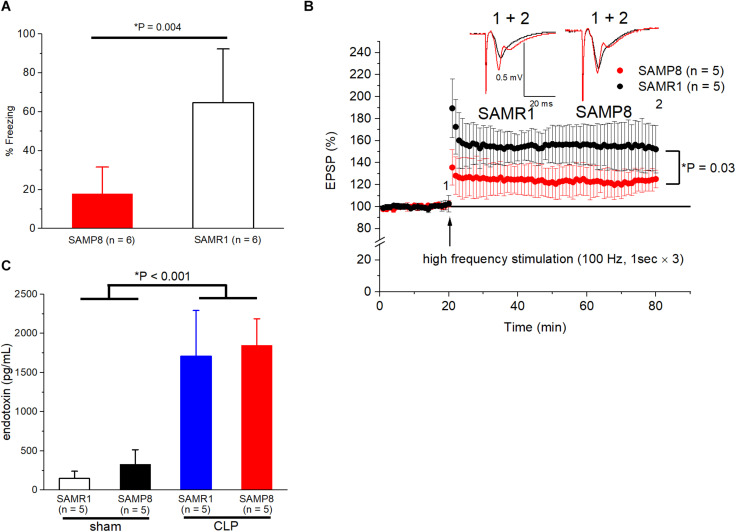
Validation of SAMP8 and CLP procedures as a mouse model of cognitive dysfunction and sepsis. **(A)** Behavioral response to trace-fear conditioning during the test session in SAMR1 and SAMP8 without surgery. A significant hippocampal-dependent memory deficit was observed in SAMP8. **(B)** Long-term potentiation in the Schaffer-collateral pathway at CA1 is impaired in SAMP8 compared to that in SAMR1. The graph shows the time course of the excitatory postsynaptic potential (EPSP) amplitudes, and representative example in panel **(B)** were sampled at the labeled time points. **(C)** Successfully induction of endotoxemia using CLP in SAMR1 and SAMP8. Data are expressed as the mean ± standard deviation. SAMP8, Senescence-Accelerated Mice Prone 8; SAMR1, Senescence-Accelerated Resistant Mice 1; CLP, cecal ligation and puncture.

First, we evaluated whether the non-surgical 6-month-old SAMP8 was an appropriate model of cognitive dysfunction. We found that the freezing time was significantly shorter for SAMP8 than that for SAMR1 in the trace fear-conditioning test (17.8 ± 13.7% vs. 64.7 ± 27.7%, *n* = 6, respectively, *P* = 0.004) (Figure 1A). Moreover, LTP was also impaired in the SC-CA1 pathway of the hippocampus in SAMP8 compared to that in SAMR1 (125.1 ± 8.1% vs. 152 ± 21.7% of the EPSP amplitude to the baseline, *n* = 5, respectively, *P* = 0.03) (Figure 1B). We subsequently measured the endotoxin concentration in the blood samples to evaluate the efficacy of CLP procedures in inducing sepsis in both mouse species. The endotoxin values revealed a significant main effect of the CLP procedure [*F*_(__1_, _15__)_ = 100.3, *P* < 0.001], no significant main effect of the mouse species [*F*_(__1_, _15__)_ = 0.55, *P* = 0.47], and no significant interaction [*F*_(__1_,_15__)_ = 0.19, *p* = 0.67] (Figure 1C), which demonstrated that CLP induced a sufficient degree of endotoxemia, irrespective of the mouse species.

### CLP Impairs SC-CA1 LTP in SAMR1, but Not in SAMP8

**FIGURE 2:**
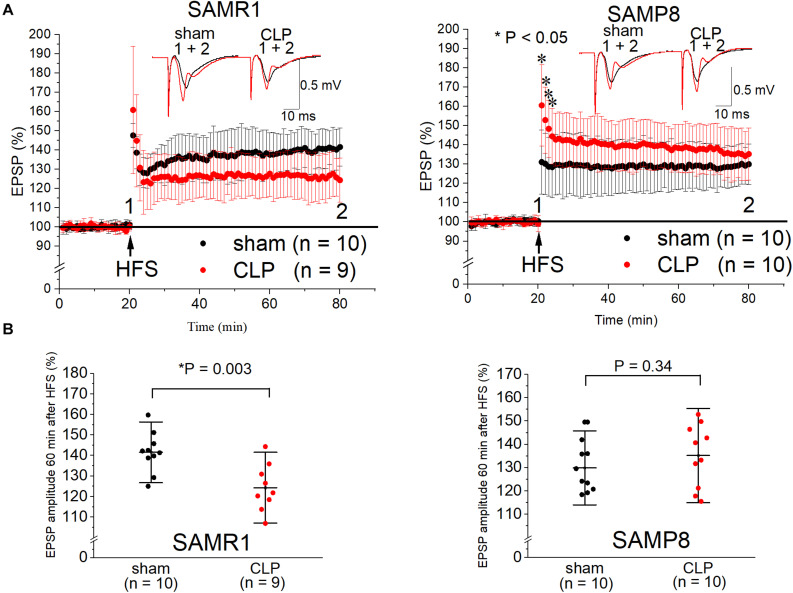
Cecal ligation and puncture (CLP) impaired Schaffer-collateral CA1 LTP in SAMR1 but not in SAMP8. **(A)** Time course of the EPSP amplitudes in SAMR1 (upper left panel) and SAMP8 (upper right panel). Although LTP was not reduced in the SAMP8 + CLP group, post-tetanic potentiation was increased 1–4 min after high frequency stimulation (HFS). Representative examples in A were sampled at the labeled time points. **(B)** Scatter plots of the percentage of the mean EPSP amplitudes obtained 60 min after LTP induction, relative to the baseline in SAMR1 (lower left panel) and SAMP8 (lower right panel). Data are expressed as the mean ± standard deviation. SAMP8, Senescence-Accelerated Mice Prone 8; SAMR1, Senescence-Accelerated Resistant Mice 1; EPSP, excitatory postsynaptic potential; LTP, long-term potentiation.

We measured the LTP at the CA1 region of the hippocampus to explore the influence of septic senescence on hippocampal synaptic plasticity in mice. The LTP in the SAMR1 + CLP group was significantly impaired compared to that in the sham-operated group 24 h after surgery (141.6 ± 9.9% vs. 124.4 ± 11.5%, *n* = 10, 9, respectively, *P* = 0.003) (Figures 2A,B). LTP was not impaired by the CLP procedure in SAMP8 (129.9 ± 10.6% vs. 135.2 ± 13.5%, *n* = 10, respectively, *P* = 0.34), although post-tetanic potentiation was significantly increased in the CLP group (131.1 ± 16.7% vs. 160.5 ± 21.2% of the EPSP amplitude 1 min after HFS, *n* = 10, respectively, *P* = 0.002) (Figures 2A,B).

### CLP Impairs Basal Synaptic Transmission and Increases Paired-Pulse Ratio in the SAMP8 Hippocampus

**FIGURE 3:**
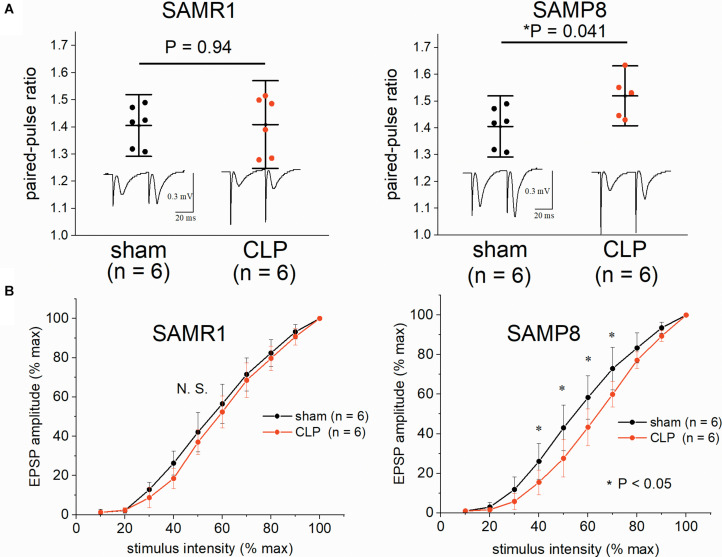
Impairment of the hippocampal synaptic basal transmission and elevation in the paired-pulse ratio in SAMP8 after cecal ligation and puncture (CLP). **(A)** CLP did not alter the paired-pulse ratio (EPSP_2_/EPSP_1_) at half-maximal intensity in SAMR1 (upper left panel), while it was significantly increased in SAMP8 (upper right panel). **(B)** The effect of sepsis on the averaged input-output curve in the hippocampi of SAMR1 (lower left panel) and SAMP8 (lower right panel). Data were normalized to the maximal-evoked EPSP and stimulus intensity range in each experiment to allow for comparison among different slices. Basal transmission was significantly reduced within the range of 40–70% of stimulus intensity in the SAMP8 + CLP group. Data are expressed as the mean ± standard deviation. SAMP8, Senescence-Accelerated Mice Prone 8; SAMR1, Senescence-Accelerated Resistant Mice 1; EPSP, excitatory postsynaptic potential.

We examined the input-output relationship and paired-pulse ratio to investigate the effect of CLP on synaptic basal release probability in the SAMR1 and SAMP8. No significant difference was observed in the paired-pulse ratio (1.41 ± 0.08 vs. 1.41 ± 0.11, *n* = 6, respectively, *P* = 0.94) (Figure 3A) and basal transmission at half-maximal intensity (42.1 ± 10.1% vs. 37.0 ± 6.3% of the EPSP amplitude to the maximal amplitude, *n* = 6, respectively, *P* = 0.31) (Figure 3B) in SAMR1. CLP significantly increased the paired-pulse ratio (1.42 ± 0.10 vs. 1.54 ± 0.07, *n* = 6, respectively, *P* = 0.041) (Figure 3A) and reduced the synaptic basal transmission (43.0 ± 9.4% vs. 27.6 ± 9.4% of EPSP amplitude at half maximal intensity, *n* = 6, respectively, *P* = 0.029) (Figure 3B) in SAMP8. The differences in basal transmission were significant within 40–70% of stimulus intensity (Figure 3B).

### CLP Elevates IL-1β Especially in the Hippocampus of SAMP8 With Microglial Activation and Is Not Correlated With Peripheral Inflammation

**FIGURE 4:**
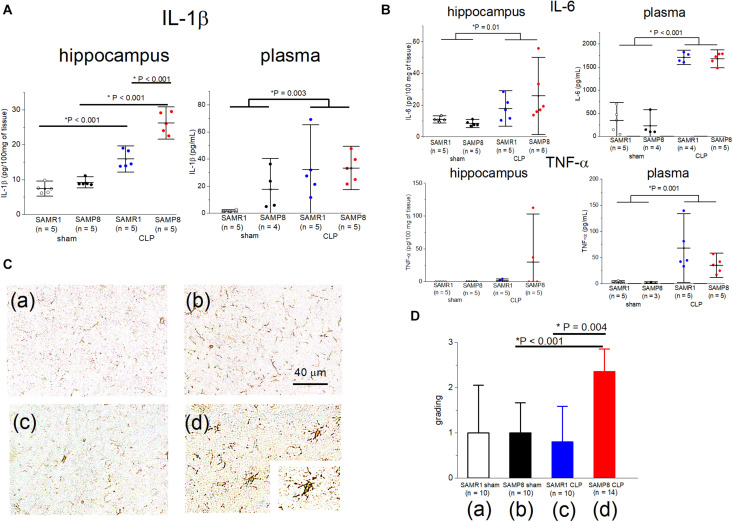
Elevation in the IL-1β concentration especially in the hippocampus of SAMP8 concomitant with microglial activation, following cecal ligation and puncture (CLP). **(A)**, Concentrations of IL-1β in the hippocampus and plasma in the four groups. There was significant interaction in the hippocampus and *post hoc* analysis revealed the significant elevation in the SAMP8 + CLP group compared to the other groups, while a slight but significant elevation was observed in the SAMR1 + CLP group compared to the SAMR1 + sham group. There was no interaction with the plasma concentration of IL-1β. **(B)** Hippocampal and plasma IL-6, and plasma TNF-α revealed the significant main effect of sepsis, but no significant interaction was observed. **(C)** Photomicrographs at the CA1 region stained with Iba-1 in the **(a)** SAMR1 + sham group, **(b)** SAMP8 + sham group, **(c)** SAMR1 + CLP group, and **(d)** SAMP8 + CLP group. The resting microglia shifted to a “reactive state” in the SAMP8 + CLP group [box **(d)**, 40×]. **(D)** Microglial activation was blindly scored on a scale of 0 (lowest) to 3 (highest). The score was significantly higher in the SAMP8 + CLP group. Data are expressed as the mean ± standard deviation. SAMP8, Senescence-Accelerated Mice Prone 8; SAMR1, senescence-accelerated resistant mice 1; EPSP, excitatory postsynaptic potential; LTP, long-term potentiation; TNF, tumor necrosis factor.

We measured the pro-inflammatory cytokine levels (IL-1β, IL-6, TNF-α) in the plasma and hippocampus to evaluate central and peripheral inflammation during the acute phase of sepsis in SAMP8 and SAMR1. The IL-1β values in the hippocampus revealed a significant main effect of sepsis [*F*_(__1_, _16__)_ = 171.4, *P* < 0.001], significant main effect of the mouse species (*F*_(__1_, _16__)_ = 38.7, *P* < 0.001), and significant interaction [*F*_(__1_, _16__)_ = 19.3, *P* < 0.001]. *Post hoc* analysis demonstrated that the value of IL-1β was significantly higher in the SAMP8 + CLP group than that in the other three groups (Figure 4A). IL-1β levels were significantly elevated in the hippocampus of CLP mice compared to the sham-operated mice in the SAMR1 group (Figure 4A). The plasma IL-1β value showed no interaction [*F*_(__1_, _15__)_ = 1.33, *P* = 0.26], although there was a significant main effect of sepsis [*F*_(__1_, _15__)_ = 12.46, *P* = 0.003] (Figure 4A). Hippocampal and plasma IL-6 levels, and plasma TNF-α levels also revealed a significant main effect of sepsis, but no interaction was observed (Figure 4B). We conducted immunohistochemical analysis with anti-Iba-1 antibodies, which are a markers of microglia, to assess the relationship between the elevation in pro-inflammatory cytokines and microglial activation in the hippocampus, *Post hoc* analysis of the findings of the four-point categorical scale (used to denote the degree of microglial activation), revealed significant activation only in the SAMP8 + CLP group compared to the other three groups [SAMR1 + sham; median 1, interquartile range (IQR) 0–2, SAMR1 + CLP; median 1, IQR 0–1.25, SAMP8 + sham; median 1, IQR 0.75–1.25, SAMP8 + CLP; median 2, IQR 2–3, *n* = 10–14, respectively] (Figures 4C,D).

**FIGURE 5:**
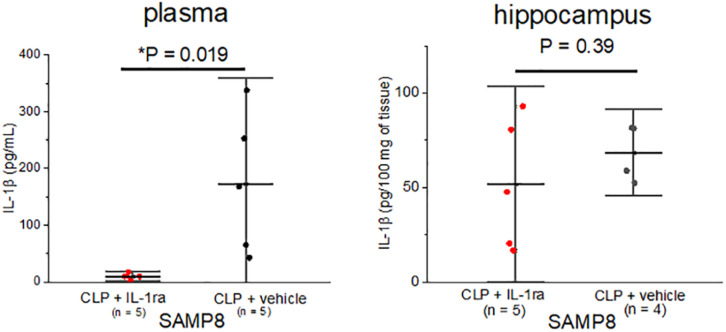
Elevation in the IL-1β concentration especially in the hippocampus of SAMP8 was not correlated with the peripheral elevation in IL-1β. Intraperitoneal IL-1ra administration reduced the concentration of IL-1β in plasma in the SAMP8 + CLP group, but that in hippocampus remained high. Data are expressed as the mean + standard deviation. SAMP8, Senescence-Accelerated Mice Prone 8; CLP, cecal ligation and puncture.

Subsequently, we examined whether the peripheral blockade of IL-1 receptors ameliorated central inflammation in the SAMP8 + CLP group. A single preemptive dose of IL-1ra decreased IL-1β concentration in plasma to nearly zero, but the hippocampal IL-1β levels remained high, despite the administration of IL-1ra (68.6 ± 15.1 pg/100 mg of tissue vs. 51.6 ± 34.5 pg/100 mg of tissue, *n* = 4, 5, respectively, *P* = 0.39) (Figure 5).

### *Ex vivo* IL-1ra Administration Ameliorates LTP Impairment in SAMR1 and Reduced Basal Transmission in SAMP8 After Sepsis

**FIGURE 6:**
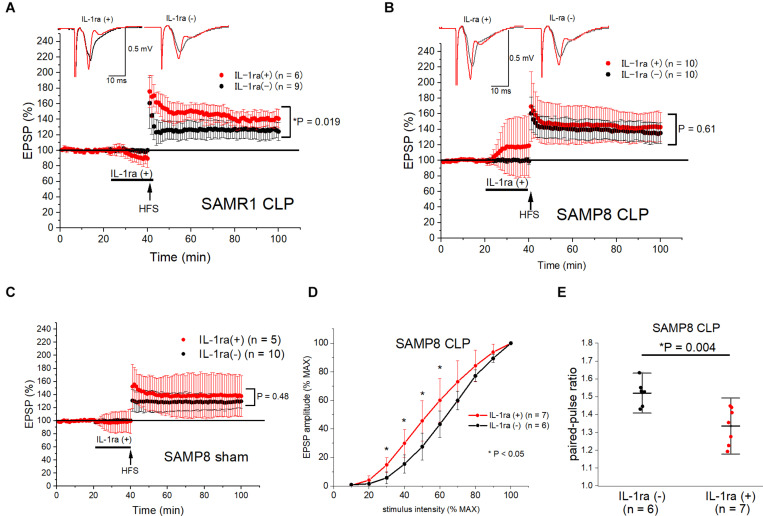
*Ex vivo* IL-1ra administration ameliorated LTP impairment in SAMR1 and the reduction in basal synaptic transmission in SAMP8 after sepsis. **(A,B)** IL-1ra perfusion for 20 min before high frequency stimulation (HFS) prevented LTP impairment in the SAMR1 + CLP group **(A)**, while it did not affect LTP in the SAMP8 + CLP group **(B)**. However, basal EPSP amplitudes were potentiated during IL-1ra perfusion in the SAMP8 + CLP group, while there was no change in the EPSP amplitudes during perfusion of IL-1ra and no significant change in LTP in the SAMP8 + sham group **(C)**. **(D,E)** Effect of IL-1ra on the averaged input-output curve **(D)** and paired-pulse ratio **(E)** in the SAMP8 + CLP group. The experiment entailed the superfusion of 100 μg/mL of IL-1ra into the hippocampal slices. The reduction in basal transmission was significantly reversed within the range of 30–60% of stimulus intensity in the IL-1ra (+) group **(D)**, and the increase in the paired-pulse ratio was also reversed **(E)**. Data are expressed as the mean ± standard deviation. EPSP, excitatory postsynaptic potential; IL-1ra, interleukin-1 receptor antagonist; SC-CA1, Schaffer-collateral CA1; LTP, long-term potentiation; SAMP8, Senescence-Accelerated Mice Prone 8; SAMR1, Senescence-Accelerated Resistant Mice 1; CLP, cecal ligation and puncture.

Finally, we perfused IL-1ra (100 μg/mL) into the acutely septic hippocampal slices for 20 min before LTP induction, to evaluate the role of IL-1β elevation in the synaptic transmission and plasticity of the hippocampi of SAMR1 and SAMP8. This concentration of IL-1ra was sufficiently high to block IL-1 receptor activation, according to a previous study (Schneider et al., 1998). We administered the IL-1ra only before HFS because low levels of IL-1β were essential for LTP maintenance (Schneider et al., 1998; Ross et al., 2003). IL-1ra administration reversed LTP impairment in the SAMR1 + CLP group (140.8 ± 12.0% vs. 124.4 ± 11.5%, *n* = 6, 9, respectively, *P* = 0.019) (Figure 6A), while it did not have any effect on the LTP in the septic SAMP8 group (142.9 ± 18.8% vs. 135.2 ± 13.5%, *n* = 10, respectively, *P* = 0.61) (Figure 6B). However, the baseline EPSP amplitude was substantially elevated during the perfusion of IL-1ra in the SAMP8 + CLP group (Figure 6B), while no such change was observed in the basal EPSP amplitude of the sham-operated SAMP8 hippocampus (Figure 6C). Therefore, we measured the input-output curve and paired-pulse ratio in the SAMP8 + CLP group at the perfused concentration of IL-1ra. We found that IL-1ra administration mitigated the reduction in basal transmission (at half-maximal intensity, 45.7 ± 14.1% vs. 27.6 ± 9.4% of the EPSP amplitude to the maximal amplitude, *n* = 7, 6, respectively, *P* = 0.02, Figure 6D) and the elevated paired-pulse ratio in the septic SAMP8 hippocampus (1.34 ± 0.10 vs. 1.52 ± 0.07, *n* = 7, 6, respectively, *P* = 0.004) (Figure 6E).

## Discussion

To the best of our knowledge, this was the first study to demonstrate the effect of sepsis on hippocampal synaptic transmission in a mouse model of aging and cognitive dysfunction, and that the IL-1 receptor-associated signals differentially modulated synaptic plasticity and basal synaptic transmission in these animals.

In our experiment, 6-month-old SAMP8 (without surgery) showed cognitive dysfunction and LTP impairment compared to the age-matched SAMR1, which was consistent with the observations of previous studies (Miyamoto et al., 1986; Taniguchi et al., 2015). CLP procedures elicited different effects on the synaptic responses of SAMP8 and SAMR1, in addition to the difference of synaptic plasticity in the basal conditions. Our results showed that LTP was impaired in the SAMR1 + CLP group, but CLP procedures did not change the hippocampal LTP in SAMP8 (Figure 2). However, increased post-tetanic potentiation, decreased input-output curve, and increased paired-pulse ratio were all suggestive of the reduction in the release probability in the SAMP8 + CLP group. The forms of short-term plasticity such as paired-pulse facilitation and post-tetanic potentiation, are associated with a reduced probability of synaptic release or increased residual levels of intracellular calcium after stimulation (Zucker and Regehr, 2002). We are aware that, ideally, an analysis of the quantal component, such as miniature excitatory postsynaptic current measurements, should be performed. However, this was difficult due to the low viability of hippocampal slices obtained from 6-month-old mice.

We speculated that these differences in the synaptic response to sepsis between SAMP8 and SAMR1 were caused by the differences in neuroinflammation. Barrientos et al. (2015) demonstrated a prolonged and remarkable elevation in hippocampal IL-1β after *E. coli* infection in healthy aging mice compared to young adult mice (Barrientos et al., 2015). Our results were consistent with their results, which showed remarkable elevation of hippocampal IL-1β levels in the SAMP8 + CLP group, while the hippocampal IL-1β levels were slightly but significantly elevated in the SAMR1 + CLP group compared to the sham-operated control group.

IL-1β is considered to be a key molecule in the mechanism underlying LTP impairment and cognitive decline in sepsis (Imamura et al., 2011). IL-1 receptors are present at high levels in the hippocampus (Parnet et al., 1994) and the sensitivity to IL-1β is augmented in aged hippocampal synapses (Prieto et al., 2015). LTP impairment in the SAMR1 + CLP group and its amelioration in response to IL-1ra administration was consistent with previous studies (Imamura et al., 2011; Hoshino et al., 2017b). This may be explained by the fact that a slight elevation in the hippocampal IL-1β levels in the SAMR1 + CLP group reduced the synaptic plasticity through the IL-1 receptor-associated signals, including the activation of the mitogen-activated protein kinase (MAPK) family (Coogan et al., 1999). However, our experiments with SAMP8 showed that LTP was not impaired despite the remarkable elevation in IL-1β during sepsis, and that basal synaptic transmission was reduced instead. Ikegaya et al. demonstrated that IL-1β induced synaptic depression in a γ-aminobutyric acid (GABA)-ergic activity-dependent manner in intact hippocampal slices (Ikegaya et al., 2003). Furthermore, Wang et al. (2012) showed that lipopolysaccharide-induced IL-1β increased the tonic inhibitory hippocampal currents through the α5-subunit-containing GABA_A_ receptors, which was associated with cognitive dysfunction after sepsis (Wang et al., 2012). Therefore, these previous studies support our findings that sepsis reduced basal synaptic transmission, concomitant with a remarkable increase in IL-1β in SAMP8 and that IL-1ra administration prevented these effects. This leads one to question the lack of impairment in LTP in the SAMP8 + CLP group. A previous study with normal rats showed that aging itself had no effect on hippocampal LTP (Kumar et al., 2007), while LTP in 6-month-old SAMP8 was inherently impaired compared to that in SAMR1. Although the mechanisms of LTP impairment in SAMP8 without surgery have not been fully elucidated, Armbrecht et al. demonstrated changes in MAPK-family associated gene expressions in SAMP8 (Armbrecht et al., 2014). Therefore, the mechanisms underlying the IL-1β-induced impairment in LTP may overlap with those of innate LTP impairment in SAMP8, and thus, the hippocampal LTP does not probably plummet any further in the SAMP8 + CLP group. Interestingly, Chapman et al. (2010) previously reported that only the theta-burst late-phase LTP was impaired in the healthy aging mouse hippocampus 4 days after *E. coli* administration, while basal transmission was unaffected (Chapman et al., 2010). These differences between healthy aging mice and senescence-accelerated mice may be crucial to the expression of cognitive dysfunction after sepsis.

Overall, the data suggest that sepsis mainly affect postsynaptic mechanisms in SAMR1 while targeting presynaptic function in SAMP8. Although IL-1 receptor type I is distributed in both presynaptic and postsynaptic terminals of the brain, it is generally enriched in the postsynaptic densities of the hippocampus (Gardoni et al., 2011). Therefore, it is reasonable that it was the postsynaptic mechanisms that were mainly affected in SAMR1 and normal adult mice, in which hippocampal IL-1β levels are slightly elevated during sepsis. Although our results lead to speculation that IL-1 receptor distribution differs between SAMR1 and SAMP8 due to the presynaptic effect in SAMP8, further studies are needed to elucidate this matter.

We found microglial activation only in the septic SAMP8 hippocampus using immunohistochemical examination (Figures 4C,D). Resting microglia usually monitor pathogens in brain’s microenvironment. The immunophenotype of microglia is altered to the “primed” state with normal aging, and the microglia are sensitized, inducing an exaggerated neuroinflammatory response to an immune challenge (Frank et al., 2010). Once activated, microglia undergo morphological changes, and produce pro-inflammatory cytokines, including IL-1β (Tanaka et al., 2006; Imamura et al., 2011). Ito et al. recently demonstrated that microglia in 17-week-old SAMP8 under physiological condition were primed and that neuroinflammatory response to lipopolysaccharide was significantly greater in SAMP8 than in SAMR1 (Ito et al., 2020). Therefore, it is reasonable that microglia were activated in the SAMP8 + CLP group in our study. However, the lack of microglial activation in the SAMR1 + CLP group, despite the slight elevation in IL-1β, was a peculiar observation. The cell types that mainly produces pro-inflammatory cytokines in the brain may differ between SAMP8 and SAMR1 because IL-1β is released by various cell types, including endothelial cells and astrocytes (Lau and Yu, 2001; Barrientos et al., 2010).

Although peripheral pro-inflammatory cytokines have been considered to be the primary initiators of neuroinflammation, our results suggested that peripheral IL-1β elevation was not necessary for neuroinflammation (Figure 5). The mechanisms that connect systemic inflammation with microglial activation remain unclear, but our results suggest that the passive influx of peripheral pro-inflammatory cytokines into the brain is not the principal mechanism. We speculate that microglia are activated via other mechanisms, such as direct activation of vagal afferents and endothelial cells (Patterson, 2015), which results in the *de novo* production of pro-inflammatory cytokines. Barrientos et al. (2015) found that the exaggerated cytokine response to the peripheral immune challenge was restricted to the brain, particularly to the hippocampus in healthy aging rats. Their findings support our results that hippocampal inflammation induced by sepsis in SAMP8 was not associated with peripheral cytokine levels.

Our study had some limitations. First, we investigated only one time-point after sepsis induction. Therefore, it is unclear whether the differences between the synaptic response in SAMP8 and SAMR1 during the acute phase of sepsis are related to the long-term cognitive outcome, i.e., SAE. However, acute cognitive decline after inflammatory events is considered to be a risk for the progression to dementia (Cunningham, 2011); thus, it is important to understand the mechanisms underlying acute cognitive decline after sepsis. Second, although SAMP8 shares some similar characteristics with human Alzheimer’s disease, they may not be the same as cognitive dysfunction in humans, warranting cautious interpretation before applying our results to clinical settings. Moreover, our study findings cannot explain the difference between the healthy elderly and those with cognitive dysfunction because we used the age-matched SAMR1 as the controls. Finally, IL-1ra also blocks the effects of IL-18 which similarly modulates synaptic plasticity in the hippocampus (Curran et al., 2003); therefore, the role of IL-18 during the acute phase of sepsis may not have been eliminated from our study.

In conclusion, this was the first study to demonstrate that synaptic transmission and synaptic plasticity are modulated differentially in SAMR1 and SAMP8 in response to sepsis, the latter of which is a mouse model of aging and cognitive dysfunction. These changes were related to the centrally derived IL-1 receptor-mediated signaling in both species of mice and were accompanied by microglial activation, especially in SAMP8. Further studies are needed to fully elucidate the mechanisms responsible for the development of SAE in the elderly and cognitive dysfunction; however, our results suggest that the development of novel therapeutic drugs for SAE should target the IL-1 receptor-mediated signaling pathway in those patients.

## Data Availability Statement

The original contributions presented in the study are included in the article/supplementary material, further inquiries can be directed to the corresponding author/s.

## Ethics Statement

The animal study was reviewed and approved by the Animal Care and Use Committee of Hokkaido University.

## Author Contributions

KH and YUchin conducted all the experiments and the statistical analysis. YUchid and HS helped the immunohistochemical analysis. KH drafted the manuscript. YM reviewed the manuscript. All authors read and approved the final manuscript.

## Conflict of Interest

The authors declare that the research was conducted in the absence of any commercial or financial relationships that could be construed as a potential conflict of interest.
